# Epigenetic Regulation of the Non-Coding Genome: Opportunities for Immuno-Oncology

**DOI:** 10.3390/epigenomes4030022

**Published:** 2020-09-10

**Authors:** Maria J. Barrero

**Affiliations:** Independent Researcher, 28049 Madrid, Spain; maria.jose.barrero@gmail.com

**Keywords:** epigenetics, immuno-oncology, repetitive elements, dsRNA, cancer, immunotherapy, transposons

## Abstract

The contribution of the non-coding genome to disease and its therapeutic potential have been largely unexplored. Recently, several epigenetic drugs developed for cancer treatment have been described to mediate therapeutic effects through the reactivation of the expression of transposable elements in cancer cells. This event activates innate immunity-related pathways and promotes the generation of neoantigens in tumor cells, improving the efficacy of immunotherapeutic treatments. This review focuses on the regulation of transposable elements by epigenetic inhibitors and its implications for immuno-oncology.

## 1. Introduction 

Traditionally, studies addressing the etiology and treatment of human diseases have focused on the role of coding genes. However, coding genes represent only 2% of the human genome [1]. Efforts to understand the contribution of the non-coding genome to disease and its therapeutic potential are beginning to emerge. Current anti-cancer strategies include the use of small molecules that function as inhibitors of enzymes able to modify DNA or histone tails. Some of these enzymes play critical roles in silencing the expression of non-coding elements of the genome. Their inhibition leads to a subsequent derepression of these elements that contributes to increase the immunogenicity of tumors. While relevant review articles on cancer epigenetics and immune-oncology have been recently published [2,3], these have mainly focused on the effects of DNA methylation inhibition or on combinatorial therapies, including ongoing clinical trials. In contrast, this review focuses on the role of epigenetics in the silencing of non-coding elements and how epigenetic drugs impact in their expression and provide opportunities to reactivate immune responses against tumors.

## 2. Immunotherapy against Cancer

**Figure 1:**
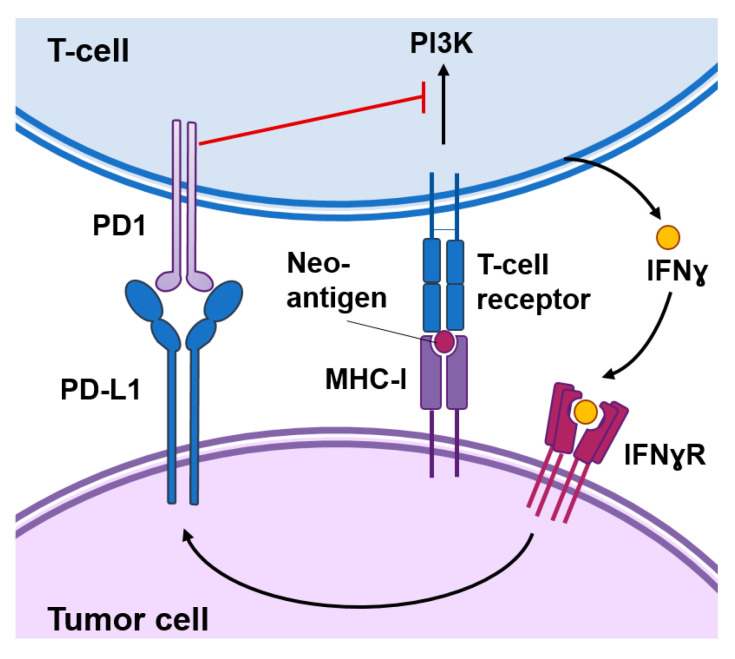
Functional interactions between cancer cells and T-cells. Cancer-specific antigens or neoantigens expressed in tumor cells processed and presented by the MHC-I system will be recognized by T-cell receptors present in the surface of T-cells. This interaction activates signaling cascades causing T-cell activation. Activated T-cells produce interferon (IFN)γ that among other responses induces the expression of the programmed death-ligand 1 (PD-L1) in tumor cells. The interaction of PD1 with PD-L1 inhibits signal transduction pathways involved in T-cell activation.

Burnet and Thomas suggested back in 1957 that the immune system has the capacity to detect and destroy abnormal cells, however it has taken several decades to develop the first successful immunotherapeutic approaches to treat cancer [4]. A key event for successful anti-tumor immunosurveillance is the expression of tumor-specific antigens or neoantigens in the cancer cell that can be recognized by the immune system and induce an immune response against the tumor. Several immuno-oncology approaches are based on the expression of these tumor-specific antigens, which might be present in the cancer cell surface or processed and presented by the major histocompatibility complex (MHC) class I molecules as immunogenic peptides (Figure 1). These approaches mainly include chimeric antigen receptor (CAR) T cells genetically engineered to recognize a specific tumor antigen or vaccines based on those antigens [5,6].

In addition, cancer cells can express modulatory signals that reinforce or prevent the immune reaction against the tumor. “Hot” tumors are typically enriched in interferon (IFN) related pathways, secrete pro-inflammatory cytokines and immuno-attractant chemokines, and are infiltrated by immune cells. However, these immune responses are not sustained for a long time as they may result in autoimmunity. Activated T cells eventually upregulate surface inhibitory receptors such as the programmed cell death protein 1 (PD-1) and the cytotoxic T-lymphocyte-associated protein 4 (CTLA-4) (Figure 1). These receptors mediate inhibitory effects by binding to their ligands in the surface of cancer cells. For example, PD-1 binds to their ligands PD-L1 and programmed death-ligand 2 (PD-L2) expressed in cancer cells in response to IFNɣ produced by immune cells. This interaction inhibits kinase-signaling pathways involved in T-cell activation leading to exhausted and dysfunctional T cells [7]. Immune checkpoint inhibitors are antibodies that block the interaction of CTLA-4 or PD-1 with their ligands and cause the reinvigoration of exhausted T cells leading to long-term favorable responses in a small percentage of cancer patients [8]. Importantly, these inhibitors function as invigorators of pre-existing anti-tumoral immune responses and “cold” tumors that express low levels of cancer-specific antigens or low levels of PD-L1 are less likely to respond [9].

Immunotherapy has achieved very good responses in a percentage of cancer patients; however, many patients have not experienced clinical benefits. Novel targets or combination with other therapies might improve these responses. Despite the traditional assumption that inhibitors of epigenetic repressors affect cancer cell growth through the reactivation of tumor suppressor genes, successful combinations of epigenetic drugs and immune checkpoint inhibitors in animal models and early clinical trials suggest that these drugs have immunomodulatory properties. Unexpectedly, the effects of these epigenetic inhibitors appear to be mediated, at least in part, through the regulation of non-coding elements of the genome.

## 3. Transposable Elements

**Figure 2:**
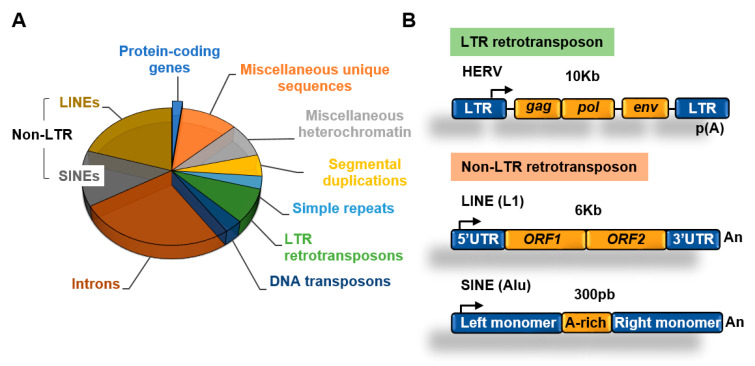
Transposable elements. (**A**) Main components of the human genome [1,11]; (**B**) structure of long terminal repeats (LTR) and non-LTR retrotransposons.

More than 50% of the human genome is made of transposable elements (TEs) which are highly repetitive DNA sequences [1] (Figure 2A). Despite their original transposition capabilities, most transposable elements present today in the human genome have lost their ability to transpose due to acquired mutations and/or epigenetic silencing. Although transposition of a reduced number of elements can play important roles in evolution and disease this topic has been covered before [10] and will not be discussed in this review. This review will focus on the role of epigenetic factors in silencing the expression of transposable elements in cancer cells and its implications for cancer therapy.

### 3.1. Retrotransposons

An abundant type of transposons are retrotransposons, which use an RNA intermediate to copy and paste themselves into genomic locations [12]. Retrotransposons, in contrast to DNA transposons that do not use an RNA intermediate for transposition, rely on cellular RNA polymerases for transcription to generate RNA. Retrotransposons include two main families depending on the presence of long terminal repeats (LTR) (Figure 2B).

#### 3.1.1. Non-LTR Retrotransposons

Retrotransposons of the non-long terminal repeat (non-LTR) category comprise long and short interspersed nuclear elements (LINEs and SINEs, respectively) [13]. The most well characterized LINE in the human genome is the LINE-1 (L1) element [14]. The human genome contains about 500,000 copies of this element, which constitutes about 20% of the genome. L1 elements are 6–7 kb long, contain a 5′ UTR region with a CpG island that acts as an internal promoter for RNA Polymerase II (Pol II), two open reading frames (ORF) that encode for proteins involved in transposition, and a 3′ UTR with a poly(A) sequence (Figure 2B).

SINEs are short elements that probably evolved from RNA genes, such as 7SL and tRNA [13]. The most abundant SINE element in the human genome is the Alu family. Alu elements are about 300 nucleotides long and constitute about 11% of the human genome. SINEs contain an RNA polymerase III (Pol III) dependent promoter and have no protein-coding capacity, relying on the activities encoded by other retrotransposons such as LINEs for transposition [15].

#### 3.1.2. LTR Retrotransposons

**Figure 3:**
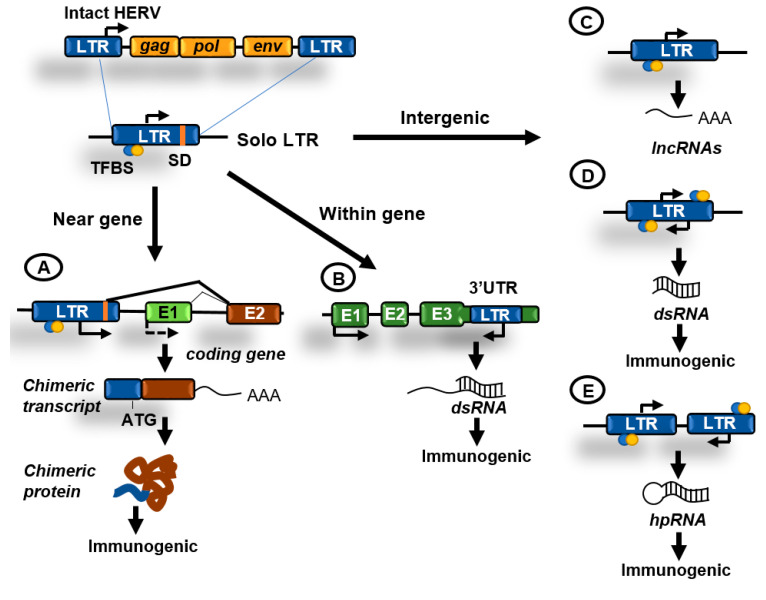
Contributions of human endogenous retroviruses (HERVs) to genome regulation. Original HERVs inserted in the human genome frequently suffered recombination between 5′ and 3′ LTR generating solo LTRs. These solo LTRs might contain transcription factor binding sites (TFBS), transcription start sites (TSS), and splicing donor (SD) sequences and might impact cellular functions in the following ways. (**A**) LTRs in the proximity of genes might function as enhancers or alternative promoters. Functioning as promoters they can generate chimeric transcripts that can be translated giving rise to potentially immunogenic proteins; (**B**) LTRs have been also located at the 3′UTR region of coding genes and might be transcribed antisense to these genes generating double-stranded RNA (dsRNA); (**C**) transcription initiated from an isolated LTR can generate long non-coding RNAs (lncRNAs); (**D**) isolated LTRs can also undergo bidirectional convergent transcription and generate dsRNA; (**E**) inverted repeats can generate hairpin RNA (hpRNA) after transcription. hpRNAs are commonly generated by inverted Alu or LINEs repeats. dsRNAs can be immunogenic by activating innate immunity pathways.

About 8% of the genome is made of LTR retrotransposons commonly represented by human endogenous retroviruses (HERVs) [1]. HERV sequences probably originated from exogenous infectious retroviruses that colonized the vertebrate and primate genomes millions of years ago. These elements originally had the ability to produce RNA transcripts that upon reverse transcription were reversed back into DNA and inserted into target sites. To carry out these functions HERVs originally contained protein-coding genes that are conservative for all retroviruses, including the gag, pro, pol, and env genes (Figure 2B). These genes are flanked by LTRs that contain transcription factors binding sites, transcription start sites (TSS) dependent on Pol II, splicing donor sites, and polyadenylation signals. At present, most of the LTR retrotransposons in the human genome are isolated LTRs, with the internal sequences being lost due to homologous recombination between flanking LTRs (Figure 3). An exception is the HERV-K family, which is the youngest and have members that possess complete open reading frames (ORFs) that can be transcribed and translated [16].

The survival of potentially dangerous retroviruses in the human genome along evolution can be partially explained by their ability to reinvent and participate in cellular functions, a phenomenon called co-option [17]. The LTR sequences can typically provide promoters, enhancers, repressors, transcription factor binding sites, polyadenylation, and alternative splicing sites for human transcripts (Figure 3A). Importantly, transposable elements have been described to drive the expression of oncogenes in human cancers acting as promoters or enhancers [18,19]. Furthermore, the biochemical activities of TE-derived proteins have been repeatedly co-opted during evolution to foster cellular innovations. The gag and env genes of HERVs have been domesticated numerous times to perform functions in placental development. The most prominent example of such domestication is the evolution of certain env genes, termed syncytins, to facilitate cell–cell fusion during placenta formation [17].

In addition, to combat the potentially harmful effects of TEs, the genome has evolved epigenetic silencing mechanisms to suppress their activity. These mechanisms include small silencing RNAs, histone and DNA modifications, as well as sequence-specific repressors such as KRAB zinc-finger proteins.

## 4. Endogenous dsRNA Production and Sensing

Although many repetitive elements are silenced by means of mutations and/or epigenetic silencing, it is now clear that some of these elements are expressed in cells under normal circumstances. The transcription of retroelements frequently gives rise to the formation of long double-stranded RNA (dsRNA) (Figure 3). Evidence suggests that dsRNAs can be produced in both healthy and cancer cells, however most studies have been done in immortalized cell lines. Most abundant dsRNAs detected in HeLa cells are generated from inverted Alu or LINEs repeats that form RNA hairpins after transcription (Figure 3E). A second less abundant source of dsRNAs is likely to be produced by bidirectional convergent transcription of HERV LTRs, which results in the production of sense and antisense transcripts and subsequent intermolecular dsRNA formation (Figure 3D) [20]. These endogenously produced dsRNA are typically longer than 100 base pairs and different from smaller silencing RNAs, such as microRNAs (miRNAs) and small-interfering RNAs (siRNAs) [16]. 

**Figure 4:**
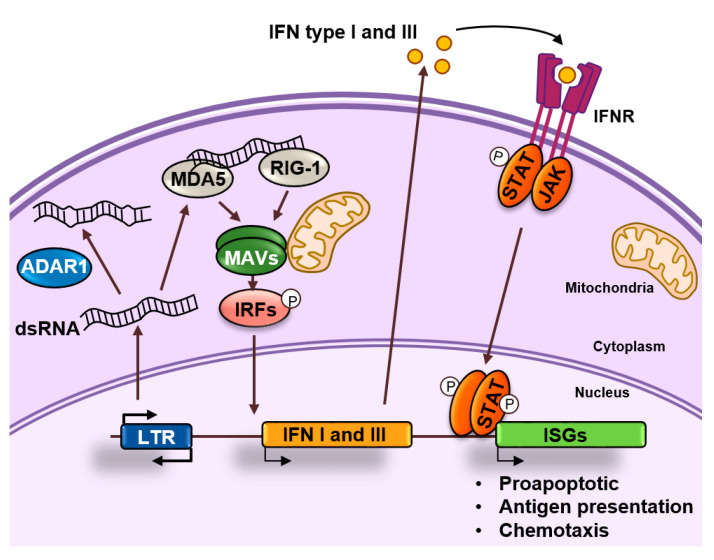
dsRNA sensing. dsRNAs accumulate in the cytoplasm as a result of retrotransposon transcription. These dsRNAs can be edited by ADAR1 or recognized by MDA5 and RIG-1 that activate and nucleate self-assembly of the mitochondrial antiviral-signaling protein (MAVS). This will result in the activation of interferon regulatory transcription factors (IRFs) that activate the expression of interferons (IFNs) type I and III. These IFNs will bind to their receptors in an autocrine or paracrine fashion and induce the expression of interferon stimulated genes (ISGs) by activating the JAK/STAT pathway. ISGs are typically involved in apoptosis, antigen processing, and presentation and chemotaxis of immune cells.

dsRNAs produced as a result of the transcription of retroelements can accumulate in the cytoplasm and activate antiviral signaling pathways in a process known as “viral mimicry” [21,22]. These dsRNAs resemble dsRNAs generated during the replication of RNA viruses and are sensed by the cell as a viral threat [23]. Long cytoplasmic dsRNAs are bound by proteins TLR3, RIG-I, and MDA5 that in turn activate the common downstream adaptor molecule MAVS (Figure 4). Activated MAVS then recruits multiple signaling molecules, including IKK-related kinases and this ultimately results in the phosphorylation of interferon regulatory transcription factors (IRFs) that activate the expression of type I and III IFNs. IFNs are secreted and bind to receptors in the producing and neighboring cells leading to the induction of an antiviral state through the upregulation of interferon stimulated genes (ISGs), which encode proteins with antiviral effector functions, including apoptosis, antigen processing and presentation, and chemotaxis of immune cells [24].

## 5. ADAR1 and the Fine Tuning of dsRNA Sensing

Cells have developed mechanisms of self-tolerance to dsRNAs consisting of their editing by the adenosine deaminases ADARs that convert adenosine to inosine (A-to-I) [25]. The conversion of A-to-I results in multiple I-U mismatches that contribute to dsRNA destabilization and prevent MDA5 activation (Figure 4) [26]. In addition, binding of ADAR proteins to dsRNA might compete with binding by sensor proteins. Among the three different ADAR enzymes, ADAR1 is responsible for most of the editing activity. ADAR1 is expressed at relatively low levels in most cells but induced by IFNs [25], that might be produced in response to dsRNA, suggesting that dsRNA editing constitutes a negative feedback mechanism that modulates the innate immunity response to dsRNAs [27].

Overall, the importance of the fine tuning of dsRNA production, editing, and detection becomes evident in diseases caused by mutations in components involved in its regulation. In humans, MDA5 gain of function mutations cause a broad spectrum of autoinflammatory diseases, including the Aicardi-Goutières syndrome [28]. Not surprisingly, this very same syndrome is caused by loss of function mutations in ADAR1 [29]. While the hyperactivation of innate immune pathways caused by these mutations is deleterious, it may offer opportunities for cancer treatment. For example, loss of function of ADAR1 in tumor cells has been reported to reduce the A-to-I editing events in dsRNAs originating from SINEs, leading to increased dsRNA sensing and tumor growth inhibition. Moreover, depletion of ADAR1 sensitized these tumors to anti-PD-1 antibody treatment [28].

## 6. Epigenetic Regulation of Retroelements

**Figure 5:**
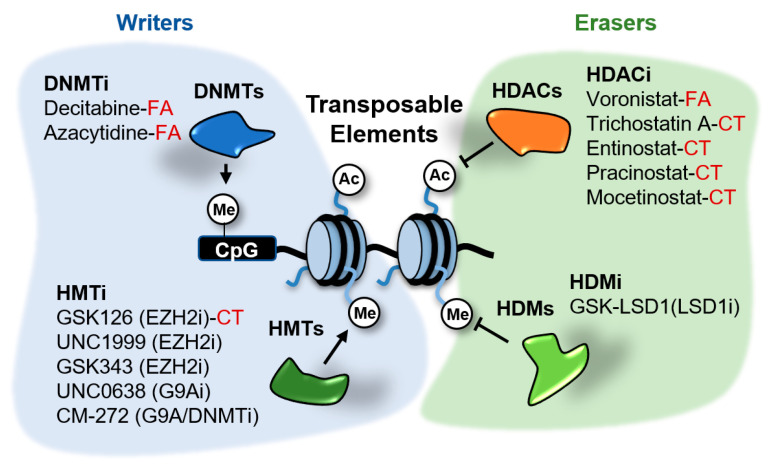
Epigenetic inhibitors described to stimulate the expression of transposable elements (TEs) in cancer cells. These inhibitors target the writers DNA methyltransferases (DNMTs) and histone methyltransferases (HMTs), and the erasers histone deacetylates (HDACs) and demethylases (HDMs). Compounds in clinical trials (CT) or FDA-approved (FA) for cancer treatment are indicated. The rest of compounds are in preclinical testing.

Different mechanisms of epigenetic silencing prevent the transcription of genomic locations. The wrapping of DNA around the histone octamers to form nucleosomes offers itself a barrier for the recruitment and processivity of RNA polymerases [30]. Highly packed nucleosomes are inaccessible for RNA polymerases and transcription factors and therefore the genes contained in these regions remain silent. Packaging of the genome is influenced by modifications in histone tails and DNA. Alterations in histone and DNA modifications are common in cancer and other diseases and therefore it is expected that small molecules able to inhibit the activity of the enzymes involved in these modifications have therapeutic potential. Several of these inhibitors are currently in clinical trials or have been FDA-approved for the treatment of certain cancers [31]. These drugs target distinct factors involved in epigenetic regulation, including writers able to modify histones or DNA, erasers that remove those marks and readers able to recognize and bind to the modifications. Several of the epigenetic enzymes targeted by these drugs, such as DNA methyltransferases (DNMTs), histone deacetylases (HDACs), the histone demethylase (HDM) LSD1/KDM1A and the histone methyltransferases (HMTs) EZH2 and G9A, play a role in the transcriptional repression of TEs in cancer cells (Figure 5). The effects of epigenetic inhibitors targeting these repressors in cancer cells will be discussed next. 

### 6.1. DNA Methyltransferases Inhibitors

DNA methylation plays a key role in gene silencing, X chromosome inactivation, genome stability, and imprinting. Although most of the interest has been focused in the role of DNA methylation in silencing the expression of tumor suppressor genes in cancer, DNA methylation is likely to play an important role in repeat element regulation since most of the genomic CpG sites subjected to DNA methylation are located at these elements [32]. 

The DNMTs inhibitors (DNMTi) azacytidine (AZA) and decitabine (DAC) are the most successful and have the longest history of epigenetic drugs used in cancer treatment to date (Figure 5). These compounds are cytidine analogs that are incorporated into DNA during replication and form covalent complexes with DNMTs depleting in this way the pool of active enzymes in the cell. AZA and DAC are currently approved for the treatment of myelodysplastic syndrome and acute myeloid leukemia. Their therapeutic effects in solid tumors appear weaker.

In addition to showing antiproliferative effects in cancer cells, DNMTi also have been described to improve the effects of immune checkpoint inhibitors in mouse models. Combinations of AZA or the HDACs inhibitor (HDACi) entinostat and anti-PD-1/anti-CTLA-4 antibodies improves the treatment outcome in mouse models of colorectal and breast cancer [33]. In clinical trials for non-small cell lung cancer (NSCLC) a small number of patients that had previously received AZA showed robust and durable responses to immune checkpoint blockade [34]. Guided by these favorable responses, clinical trials are on course to determine if DNMTi can sensitize patients with different types of cancer to immune checkpoint inhibition.

More mechanistic studies revealed that cancer cell lines treated with low doses of AZA induce the expression of genes involved in innate immunity, including antigen presentation, apoptosis, interferon signaling, and anti-viral defense [34,35]. Deep investigation into the molecular mechanisms responsible for the induction of innate immunity genes and potential benefit in combination with immunotherapy, revealed the accumulation of cytosolic dsRNA that triggers a type I interferon response after treatment of ovarian and colorectal cancer cell lines with DNMTi [21,22]. These dsRNAs were likely originating from increased expression of multiple DNA hypermethylated HERVs.

Fine mapping of transcriptional responses to DNMTi in the lung cancer cell line NCI-H1299 revealed that treatment with DAC had no significant direct effects on gene promoter driven transcription. Instead it induced the transcription of thousands of non-annotated transcription start sites (TSSs) that overlapped with TEs [36]. The most enriched elements experiencing non-annotated transcription initiation after DAC treatment were solo LTRs belonging to the LTR12C family. Combinations of HDACi (vorinostat or pracinostat) and DAC had the most prominent effects in inducing the expression of these elements. Loss of DNA methylation likely facilitated the binding of transcription factors like GATA2 to these elements, inducing histone acetylation and consequent engagement in transcription. Surprisingly, the LTRs induced by the treatments displayed unidirectional transcription and it is unclear how they might contribute to generate dsRNAs. Interestingly, those elements were found also enriched in H3K9me3 suggesting that they could be also induced by inhibitors of writers of this mark.

In addition to the effects of DNMTi in stimulating innate immunity pathways these inhibitors might also enhance the effects of immune checkpoints inhibitors through the induction of expression of tumor-specific antigens [34,35]. Recent evidence suggests that the induction of TEs expression contributes to generate immunogenic peptides. First, in the NCI-H1299 cancer cell line DNMTi induces the expression of several LTRs located in the proximity of coding genes generating fusion transcripts that encode novel protein isoforms [36] (Figure 3A). These chimeric proteins, partially encoded by repetitive elements, might become immunogenic if processed and presented by cancer cells. Second, glioblastoma cell lines can express and present several neoantigens derived from TEs after treatment with AZA [37]. These potentially immunogenic peptides are derived not only from HERVs, but also from other classes of TEs including LINEs and SINEs and might not only contribute to stimulate immune responses against tumors but could be used to develop personalized anti-cancer vaccines. Therefore, DNMTi might offer combinatorial opportunities with different immuno-oncology strategies, including immune checkpoint inhibitors, vaccines, and perhaps CAR-T cells targeting peptides originating from TEs.

A major drawback of epigenetic therapies that has hampered their progress in the clinic is the inability to select which cancer patients are more likely to respond to therapy. Finding novel markers to predict sensitivity to DNMTi might help to find clinical applications for these drugs in solid tumors. Several lines of evidence suggest a correlation between the expression of TEs, enrichment in innate immunity-related gene signatures and sensitivity to DNMTi treatment. Chiappinelli et al. observed that ovarian serous cancers with high HERV expression were enriched in innate immunity signatures [21]. Additionally, enrichment of innate immunity signatures correlated with sustained response to immune checkpoint inhibitors in melanoma patients. In a similar way, Kong et al. described that tumors with great loss of DNA methylation at TEs showed TEs reactivation and presence of immune infiltrates [37]. Finally, low LINE-1 expression has been correlated with global DNA hypermethylation and responsiveness to AZA in colorectal cancer cell lines [38]. This raises the idea that tumors with low innate immunity signature and low HERV expression might benefit from DNMTi treatment to reactivate expression of TEs and promote anti-tumor immunity. Therefore, the level of expression of certain repeats, their methylation status, or the enrichment in innate immunity signatures in tumors could be used as a predictor of response to DNMTi.

### 6.2. LSD1 Inhibitors

Histone lysine specific demethylase LSD1 (KDM1A) was the first discovered histone demethylase [39]. LSD1 was characterized as a transcription co-repressor that works primarily by demethylating mono and dimethylated lysine 4 of histone H3 (H3K4me1/2) [40]. In very specific cases LSD1 might also display H3K9 demethylation activity contributing to gene activation [41]. In addition LSD1 can demethylate a number of non-histone proteins [42].

LSD1 has been described to be involved in several types of cancer. High LSD1 expression correlates with malignancy in a range of solid tumors [43,44,45]. In distinct solid tumors and hematologic malignancies LSD1 inhibits differentiation, and enhances proliferation and invasiveness [43,46]. Accordingly, inhibitors of the catalytic activity of LSD1 (LSD1i) have shown antiproliferative effects in preclinical models of cancer and are being tested in clinical trials [42,47,48,49,50,51].

Having LSD1 multiple targets and acting both as a coactivator and corepressor has complicated the understanding of how LSD1i block proliferation of cancer cells. Inhibition of transcriptional programs governed by oncogenic transcription factors and induction of differentiation pathways have been reported in solid cancer cell lines and mouse models of hematologic cancers [46,52]. Moreover, several studies report that LSD1i treatment can stimulate immune responses against tumors. LSD1i induced the expression of cytotoxic T cell attracting chemokines in triple negative breast cancer (TNBC) cell lines [53]. In a mouse model of breast cancer combinations of LSD1i with anti-PD-1 antibodies significantly suppressed tumor growth and pulmonary metastasis, and increased T cell infiltration [53]. In pediatric high-grade glioma (pHHG) cell lines LSD1i induced an immune-related gene signature and promoted tumor regression and augmented natural killer (NK) cells infiltration of tumors in a mouse model of this disease [54].

Recent data suggests that LSD1i might potentiate anti-tumor immune effects through the reactivation of HERVs. Treatment of cancer cell lines with the LSD1i GSK-LSD1 resulted in the induction of interferon-related genes and expression of a subset of HERVs in several cancer cell lines [55]. While interferon-related genes were not direct targets of LSD1, certain HERVs were found occupied by LSD1, gained H3K4 methylation, and were induced after LSD1 depletion. Sense and antisense transcripts originating from these HERVs as well as accumulation of dsRNAs could be observed in response to LSD1 knock down. As expected, the responses to LSD1 depletion were dependent on of TLR3 and MDA5. Furthermore, authors demonstrated that inhibition of LSD1 in cancer cells increased their immunogenicity. Inoculation of wild type and LSD1 depleted B16 cells into immunocompetent mice showed that LSD1 ablation reduced tumor growth and promoted T cell infiltration into tumors in an MDA5 dependent fashion. Further combination of LSD1 depletion with PD-1 blockade showed striking anti-tumor effects.

Analysis of gene expression data in cutaneous melanoma, TNBC, and pHHG patients revealed inverse correlations between LSD1 expression and CD8+ T cell infiltration, prognosis, levels of cytotoxic T cell attracting chemokines expression, PD-L1 levels, and innate immunity signatures [53,54,55]. These data suggest that LSD1 expression may be an informative biomarker for prognosis and treatment.

From the mechanistic point of view there are still many unresolved questions about the effects of LSD1i in the activation of TEs. A fine dissection of the sites of cryptic transcription induced by these inhibitors is granted to fully understand their effects. This will allow to determine whether DNMTi and LSD1i regulate the same or different types of elements and whether inhibition of both will further stimulate anti-tumor immunity. In addition, further work is needed to explore the effects of LSD1 inhibition in immune cells.

### 6.3. EZH2 Inhibitors

EZH2 is the catalytic subunit of the PcG repressor complex 2 (PRC2) that trimethylates lysine 27 on histone 3 (H3K27me3), a mark involved in gene repression. Several lines of evidence have implicated EZH2 in the development and progression of a variety of cancers. EZH2 overexpression has been shown to correlate with aggressiveness and advanced disease in several cancer types [56,57,58]. Additionally, gain of function mutations that increase the catalytic activity of EZH2 have been described in follicular lymphomas, diffuse-large B cell lymphomas, and melanomas [59,60,61,62]. Given the evidence for EZH2 involvement in cancer, the development of EZH2-specific catalytic inhibitors (EZH2i) has been an active area of investigation for quite some years. A major current goal is the search for molecular markers predictive of patient response. In this regard, EZH2i have been reported to cause antiproliferative effects in several cancer cell lines including lymphoma cell lines with EZH2-activating mutations [63,64,65] and cancer cell lines with inactivating mutations in subunits of the chromatin-remodeling complex SWI/SNF such as ARID1A-deficient ovarian cancer [66], SMARCA4- and SMARCA2-deficient ovarian cancer [67], and SMARCB1/INI1-deficient rhabdoid tumor and synovial sarcomas cell lines [68,69]. While several clinical trials are testing these potential dependencies in patients, early in 2020 the EZH2i tazemetostat received for the first time FDA approval for the treatment of epithelioid sarcomas with SMARCB1/INI1 deletions.

In addition to the described antiproliferative effects, several studies have shown that the treatment with EZH2i improves the responses to diverse immunotherapy approaches in mouse models of ovarian, bladder cancer, and melanoma [70,71,72] and several clinical trials are taking place to test the efficacy of such combinations in cancer patients [73]. Among the molecular mechanisms responsible for such actions EZH2 has been described to be involved in regulating both cancer cell immunogenicity, through the silencing of immuno-attractant cytokines and antigen presentation-related genes, and T cell anti-tumor responses [70,71,72,74].

Recent evidence suggests that EZH2 plays a role in silencing TEs in cancer cells. Lower levels of EZH2 and higher levels of innate immunity-related genes expression have been reported in chemotherapy-resistant small-cell lung cancer (SCLC) cell lines compared to parental sensitive cell lines [75]. Reduced expression of EZH2 in resistant cell lines caused antisense transcription of several HERVs located in the 3′UTR regions of ISGs that generated dsRNAs by paring the sense transcripts generated from ISGs in response to IFNs (Figure 3B). Similar results were obtained after treatment of parental cell lines with the EZH2i GSK-126. Another study showed that taxane-resistant TNBC cell lines had reduced levels of metabolites of the methionine cycle and decreased S-adenosyl-L-methionine (SAM) production compared to parental sensitive cell lines [76]. These metabolic changes correlated with decreased levels of DNA methylation at TEs and a relocation of the H3K27me3 mark to these regions to maintain TEs repressed. Treatment with EZH2i UNC1999 or GSK343 induced the expression of HERVs and accumulation of dsRNA in taxane-resistant cell lines but not in the parental sensitive lines. Importantly, a similar epigenetic switch from DNA methylation to H3K27 trimethylation at TEs has been also described in mouse embryonic stem cells (mESCs) cultured under DNA-hypomethylating conditions [77]. These results illustrate how the environment can change the epigenetic mechanisms silencing the expression of TEs and create new vulnerabilities.

### 6.4. HDAC Inhibitors

Histone acetylation plays an important role in gene expression. Acetylated histones are recognized by epigenetic readers that participate in the recruitment of Pol II to gene promoters. Levels of acetylation at particular genomic locations are the result of an equilibrium between histone acetyl transferases (HATs) and histone deacetylates (HDACs). There are 18 human HDACs grouped into four classes based on their primary homology to yeast HDACs. Among these, class I and II HDACs play a major role in the lysine deacetylation of N-terminal histone tails. Class I comprises HDAC1, 2, 3, and 8. Class II is further divided into two subclasses: IIa (HDAC4, 5, 6, 7, and 9) and IIb (HDAC6 and 10). Four inhibitors (vorinostat, romidepsin, belinostat, and panobinostat), which target several HDACs among the different classes, have been FDA approved for the treatment of several hematological malignancies [78]. Other HDACi such as entinostat, are currently undergoing clinical trials (Figure 5).

Evidence obtained in clinical trials suggest that treatment with HDACi improves the efficacy of immunotherapeutic approaches [79]. Effects of HDACi might be exerted at several levels of the tumor microenvironment. In cancer cells, HDACi induce the reactivation of genes involved in antigen presentation and the production of immunomodulatory chemokines [79]. As discussed above, recent data suggest that these effects might be at least partially mediated by the reactivation of endogenous retroviruses. Both DNMTi and HDACi stimulated cryptic transcription of LTR12C retroviral elements in the lung cancer cell line NCI-H1299 [36]. Induction of LTR12 transcription by HDACi mocetinostat and entinostat that target specifically HDACs 1, 2, and 3 was also reported in cancer cells derived from many tumor species [80]. However, in this study authors suggest that the targeted LTR12 elements act as promoters that drive the expression of proapoptotic genes after HDACi treatment rather than a source of dsRNAs. The effects of HDACi on TEs might not be limited to cancer cells since exposure of primary CD4+ T cells to a high dose of vorinostat has been reported to also induce expression of LTR12 elements [81]. 

### 6.5. G9A Inhibitors

G9A is a mammalian histone methyltransferase that catalyzes the methylation histone H3 at lysine 9 (H3K9), participates in gene repression, and is required for the establishment of the silencing of newly integrated proviruses in mESC [82]. G9A is frequently overexpressed in cancer and correlates with poor prognosis. In agreement, blocking its methyltransferase activity has been reported to block the proliferation of certain cancer cell lines [16]. Several inhibitors of G9A catalytic activity (G9Ai) have been developed, however, their potential for clinical application is limited by their poor pharmacokinetics in vivo. 

Despite being less explored than the previously discussed inhibitors, combination of G9Ai and DNMTi caused synergistic antitumor effects that correlated with the upregulation of HERVs and viral defense genes in ovarian cancer cell lines with high levels of G9A expression [83]. HERVs upregulated by G9Ai or DNMTi alone were mostly different being CpG-rich HERVs more likely to be repressed by DNA methylation [83,84]. Surprisingly, a large number or HERVs were induced only by the combination of both treatments. This might be explained by an epigenetic switch in which these HERVs become repressed by G9A-H3K9 mediated methylation upon loss of DNA methylation. In addition, different ovarian cancer cell lines responded upregulating different HERVs and no correlation was found between the number of HERVs upregulated and the magnitude of induction of viral defense-related genes. These findings suggest that the mechanisms that repress HERVs are to some extent cell specific and that not all HERVs are equal with respect to their abilities to activate the viral defense pathway. In a similar way, a recent study described the upregulation of HERVs, accumulation of dsRNAs, and induction of innate immunity-related genes by the dual G9a/DNMT inhibitor CN-272 in bladder cancer cell lines. Treatment with this inhibitor improved the antitumor effects of anti-PD-L1 therapy in a transgenic mouse model of aggressive metastatic, muscle-invasive bladder cancer [85].

In addition to G9A, other H3K9 methyltransferases have been involved in the repression of HERVs. For example, the methyltransferase SETDB1 is known to play a role in the silencing of retroelements in mESCs [82]. Knock out of SETDB1 in acute myeloid leukemia (AML) cell lines triggered the expression of retrotransposable elements, production of dsRNAs, and the activation of viral defense-related genes [77]. These results are in agreement with the described function of SETDB1 as a corepressor of transcription factors KRAB-ZFPs that play a major role in the recognition and transcriptional silencing of transposable elements [86,87].

## 7. Perspectives

Repetitive elements have been considered “junk” DNA for a long time and the study of their regulation and expression largely disregarded. In the last decade, the contribution of transposable elements to the etiology of diseases and their potential to treat cancer is becoming clear. As a field that is emerging, still many critical questions remain unanswered.

The evolutionary complexity of retrotransposons suggests that alterations in the epigenetic silencing of these elements might have multiple consequences. In addition to a source of non-coding transcripts and dsRNAs, co-opted HERVS have been described to drive the expression of both oncogenes and tumor suppressors [19,88]. In addition, certain HERVs can provide binding sites for IFN-inducible transcription factors, such as STAT1 and/or IRF1, in response to IFN treatment [89]. Given this complexity, fine mapping of the sites of cryptic transcription induced by epigenetic drugs will be needed to fully understand their mechanism of action. Additionally, a characterization of the produced dsRNAs and their ability to activate the cellular viral defense pathways is granted. It is also important to consider that inhibitors of epigenetic repressors have been largely involved in the reactivation of tumor suppressors controlled by cellular promoters and enhancers which might still significantly contribute to the antiproliferative effects mediated by these drugs. At the end, these drugs might exert their antiproliferative effects through multiple mechanisms that might vary from tumor to tumor. Understanding these mechanisms can have critical consequences for the efficacy of therapies. While effects mediated by tumor suppressors hold therapeutic potential on their own, the activation of innate immunity pathways calls for combinations with immunotherapies.

Another question is to which extent the reactivation of different subfamilies of TEs in response to epigenetic drugs is tumor or cell specific. Activation of the same LTR12C elements by DNMTi and HDACi treatment but not by other epigenetic inhibitors has been seen in different cancer cell lines [90]. However, little is known about the sensitivity of particular subfamilies of TEs to inhibitors of other epigenetic factors. The fact that not all cancer cell lines are sensitive to the action of these inhibitors suggests that induction of TEs could be cell specific. Examples of differential sensitivity based on the expression levels of epigenetic enzymes or metabolic abundance of their cofactors have been described [76,83]. A critical question is how much the cancer cell line transcriptional program is impacting the epigenetic status of these elements that could make them more or less susceptible to the distinct inhibitors. Can this sensitivity be correlated with cancer driving mutations? Importantly, mutations in signal transduction pathways have been described to be able to impact the epigenome of cancer cells broadly [77]. An alarming fact is the possibility that these drugs mediate reactivation of TEs in normal cells, which might contribute to potential toxicity.

Fine dissection of the mechanisms involved in the response to epigenetic drugs is needed to select potential responsive patients as well as successful combinatorial therapies in clinical trials. The expression of TEs in tumors is associated with immune infiltration and increased antigenicity [37]. Reactivation of these elements using epigenetic drugs can help to turn “cold” tumors into “hot” tumors. Therefore, tumors with low TEs expression or poor enrichment of IFN-related signatures might be potential candidates for therapy using epigenetic inhibitors. A fundamental question is which class of inhibitor will show higher efficacy in each tumor and what sort of responses will be predominant. Reactivation of innate immunity- and IFN-related pathways might benefit from combinations with immune checkpoint inhibitors while induction of the expression and presentation of viral proteins or neopeptides originating from TEs might benefit from personalized anti-cancer vaccines based in these epitopes.
